# Global epidemiology of prediabetes - present and future perspectives

**DOI:** 10.1186/s40842-019-0080-0

**Published:** 2019-05-09

**Authors:** Ulrike Hostalek

**Affiliations:** Global Medical Affairs, Merck KGaA, Frankfurterstr. 250, 64293 Darmstadt, Germany

**Keywords:** Prediabetes, Diabetes, Global burden, Prevalence, Screening tools

## Abstract

Prediabetes is defined as an intermediate state of hyperglycaemia with glucose levels above the normal state but below the diagnostic levels of diabetes. It is increasingly recognised as an important metabolic state, as individuals with prediabetes are at high risk of developing overt diabetes and its associated complications. A better understanding of prediabetes could help with earlier identification, thereby allowing earlier intervention, potentially lowering the number of individuals who go on to develop diabetes. The definitions and screening criteria for prediabetes differ between guidelines published by different organisations, resulting in estimations of prevalence that can vary widely from one another. Despite these differences, these estimates suggest that the number of individuals affected by prediabetes is increasing rapidly in all areas of the world. This short review compares and contrasts the diagnostic criteria for screening of prediabetes, the impact of various glycaemic measures on prevalence estimates, and discusses current and future trends in the global prevalence estimates of prediabetes.

## Background

Prediabetes is increasingly recognised as an important metabolic state; as well as predisposing individuals to a high probability of future progression to diabetes, individuals with prediabetes are at increased risk of developing many of the pathologies normally associated with that disease, such as diabetic retinopathy, neuropathy, nephropathy and macrovascular complications [1].

In a cohort of individuals from the Diabetes Prevention Program (DPP), who were at high risk for developing diabetes, the prevalence of diabetic retinopathy was 7.9% [2]. In a different study, the prevalence of peripheral neuropathy was higher in those with prediabetes than in those with normal glucose tolerance, and was similar to that in participants with recently diagnosed diabetes [3]. An association between prediabetes and increased risk of chronic kidney disease (CKD) has also been reported, based on results from a meta-analysis [4]. Another meta-analysis showed that, compared with normoglycaemic individuals, there is an increased risk of cardiovascular disease, coronary heart disease, stroke and all-cause mortality in those with prediabetes [5]. In addition, elevated plasma glucose levels indicative of prediabetes in early pregnancy are associated with increased risk of adverse pregnancy outcomes, and may also lead to gestational diabetes in later pregnancy [6].

Besides these associated complications, the view that prediabetes is a distinct pathological condition to diabetes is supported by the recent inclusion of codes specifically listing ‘prediabetes’ as a separate billable condition in the International Classification of Diseases, 10th Revision, Clinical Modification (ICD-10-CM) [7].

Prediabetes will progress to overt type 2 diabetes (T2DM) in approximately 25% of subjects within 3–5 years, and as many as 70% of individuals with prediabetes will develop overt diabetes within their lifetime [1, 8]. As a chronic disease, the long-term implications of diabetes contribute to poor quality of life and greatly increase healthcare expenditure [9]. Prediabetes may however be reversible, through the implementation of lifestyle modification programmes based around the adoption of healthier diet and increased levels of physical activity [10, 11]. Where lifestyle modifications are ineffective, medications such as metformin or acarbose may be indicated [10, 11]. A commonly cited statistic states that 1:3 Americans has prediabetes, and that 90% are not aware of their condition, [12] but how does this compare to estimates derived from other studies conducted in other countries? This short review assesses the current and future trends in the global prevalence of prediabetes.

## Defining prediabetes

Prediabetes is a chronic metabolic condition where blood glucose levels are above the upper threshold considered normal but below the threshold for a diagnosis of diabetes [1, 8]. Importantly, the diagnostic criteria and terminology associated with prediabetes vary considerably between organisations, and care must be taken when interpreting and describing prevalence and incidence data (Table 1) [7, 9, 13].

**Table 1:** Diagnostic criteria for prediabetes

Criterion	ADA [13]	WHO [7]	IDF [9]
Terminology	Prediabetes	Intermediate hyperglycaemia	Impaired glucose tolerance
IGT (assessed using 2-h PG during 75 g OGTT)	7.8–11.0 mmol/L (140–199 mg/dL)		
IFG (assessed using FPG)	5.6–6.9 mmol/L (100–125 mg/dL)	6.1–6.9 mmol/L (110–125 mg/dL)	
HbA_1C_	5.7–6.4% (39–47 mmol/mol)	ND	

ADA, American Diabetes Association; FPG, fasting plasma glucose; HbA_1C_, glycated haemoglobin; IDF, International Diabetes Federation; IFG, impaired fasting glucose; IGT, impaired glucose tolerance; ND, not defined; OGTT, oral glucose tolerance test; PG, plasma glucose; WHO, World Health Organization.

Both the World Health Organization (WHO) and the American Diabetes Association (ADA) provide guidance on screening for prediabetes based on assessment of impaired glucose tolerance (IGT) and impaired fasting glucose (IFG) levels. While the defined thresholds for IGT are common to both guidelines, the ADA recommend a lower threshold for IFG relative to the WHO guidelines. [7, 13] This move was an attempt to improve the concordance of prevalence estimates between IFG and IGT, which when defined using WHO thresholds can differ considerably from each other. The criteria used by the WHO were derived not on overall prevalence, but to reflect the relative likelihood of progression to overt T2DM; IFG, reflective of hepatic insulin resistance is considered the more important predictor of diabetes risk than skeletal muscle insulin resistance described by IGT [14].

IFG is assessed based on the fasting plasma glucose (FPG) level, and IGT using the 2-h plasma glucose during a 75 g oral glucose tolerance test (OGTT). The ADA have also recommended the assessment of glycated haemoglobin (HbA_1c_) to screen for prediabetes, [15, 16] however, this view is not endorsed by the WHO. These tests do not necessarily detect prediabetes in the same individuals, and according to ADA guidance, abnormal results from any of the tests is sufficient for prediabetes diagnosis. HbA_1C_ is considered by many as a more reliable test of impaired glucose homeostasis as it is reflective of steady-state blood glucose levels over a period of several months, so is not prone to day-to-day variability that may confound assessment of IGT or IFG [15]. It is also a more convenient screening test than FPG or OGTT, as fasting is not required [13]. However, the availability of HbA_1C_ testing may be limited in developing countries [13]. As an indirect measure of average blood glucose levels, HbA_1C_ may be affected by ethnicity and haemoglobin variants, [13, 17] although most assays used in the US are unaffected by common variants [13]. In the DPP, African Americans had higher HbA_1C_ levels than Caucasians, despite similar fasting and 2-h glucose levels [18]. In addition, it is worth noting that key diabetes prevention studies, such as the DPP and its long-term follow-up Diabetes Prevention Program Outcomes Study (DPPOS), which enrolled patients at high risk of developing diabetes, did not use HbA_1C_ as an inclusion criterion [19, 20]. When DPP was initiated, HbA_1C_ was not an established measure of glycaemic control; however, additional analyses of the data have since been conducted, and baseline HbA_1C_ was shown to be a strong predictor of diabetes incidence [21].

It is unclear which diagnostic criteria are the most appropriate for identifying people with prediabetes, but as mentioned above, the lower threshold for IFG in the ADA guidelines compared with the WHO and the International Diabetes Federation (IDF) guidelines improves the parity between the number of people identified by IFG and IGT. The ADA recommendations are regularly updated with the most recent scientific data and may be considered more globally accepted than the other guidelines.

With a range of criteria available for prediabetes identification, it is not surprising that populations with prediabetes identified by each method vary widely and have limited overlap [17]. These differences in screening criteria for prediabetes may result in incorrect diagnoses, leading to some people being unnecessarily treated, and others left without treatment to prevent or delay the onset of overt type 2 diabetes. Similarly, it is difficult to assess the global burden of prediabetes.

## Prevalence of prediabetes

Prevalence estimates of prediabetes reported in the literature vary greatly, due to the diagnostic criteria used, the choice of test and due to the populations being studied (Table 2). The lower cut-off defined by the ADA guidelines lead to much higher prevalence rates compared with those defined by WHO guidelines; in a cohort of 1547 American adults without diabetes, changing the lower IFG threshold from 110 mg/dL to 100 mg/dL resulted in an increase in prediabetes prevalence from 19.8 to 34.6% [22]. A large meta-analysis of studies that reported prevalence in Caucasian and Asian cohorts estimated IFG prevalence at 36.0% using WHO guidelines and 53.1% using ADA guidelines. By contrast, the same meta-analysis described similar prevalence rates for patients who have both IFG and IGT; 15.8% for WHO and 20.2% for ADA guidelines [14].

**Table 2:** Variation in estimated prediabetes prevalence in the literature

Author	Guideline criteria used	Estimated prevalence (%)					
		IFG or IGT	IFG	IGT	IFG and IGT	HbA_1C_	HbA_1C_ and IFG
Karve [22]	ADA^a^	19.8	4.5	11.8	3.5	–	–
	ADA†	34.6	19.4	5.4	9.8	–	–
Yip [14]	ADA†	–	53.1	23.8	20.2	–	–
	WHO	–	36.0	45.5	15.8	–	–
Blum [23]	ADA†	–	3.0	–	–	24.7	3.2

^a^1997 guidelines, FPG: 6.1–6.9 mmol/L (110–125 mg/dL); †post 2003 guidelines, FPG: 5.6–6.9 mmol/L (100–125 mg/dL)

*ADA* American Diabetes Association, *FPG* Fasting plasma glucose, HbA_1C_, glycated haemoglobin; *IFG* Impaired fasting glucose, *IGT* Impaired glucose tolerance, *WHO* World Health Organization

As mentioned above, HbA_1C_ is considered by many as a more reliable test of impaired glucose homeostasis. A study of 1542 healthy Swiss adults identified prediabetes in 30.9% of the population; of these 79.9% were identified on the basis of HbA_1C_, 9.9% on the basis of FPG, and 10.3% on the basis of both of these ADA guideline criteria (HbA_1C_ and IFG) [23].

As would be expected, prevalence estimates vary widely depending on the diagnostic test used, even within the same study population. In some cases, prediabetes diagnosis is based on one criterion (e.g. IGT), while others evaluate results from more than one test. According to the ADA guidelines, an abnormal finding of any of the three criteria (IFG, IGT and HbA_1C_) is sufficient to confirm prediabetes [13]. Relying on one test may underestimate prevalence.

## Global variability in prediabetes prevalence

**Fig. 1:**
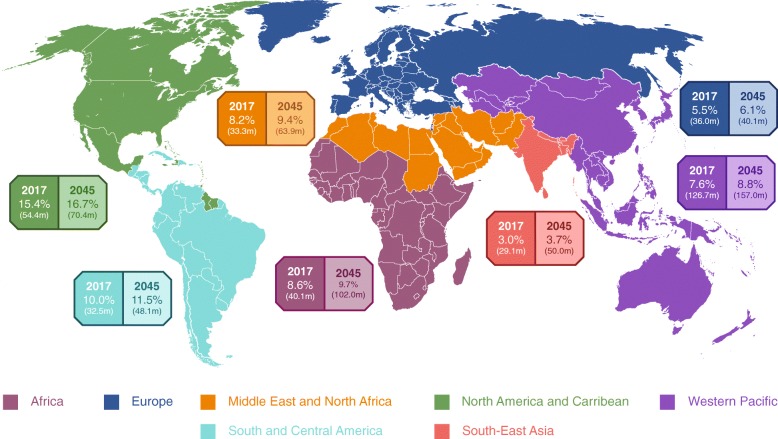
Global prevalence estimates of IGT by IDF region [9]. Data source: IDF Diabetes Atlas – 8th Edition. Percentages represent unadjusted regional prevalence estimates. Numbers in parentheses represent the estimated number of individuals affected by IGT in each region. Prevalence estimates calculated by the IDF using a generalised linear regression model. A variety of country-level data sources were included, mostly from peer-reviewed journals and national health surveys. The studies selected were required to meet rigorous inclusion criteria based on input from international experts. Prevalence in countries without original data available was extrapolated based on data collected from similar countries, based on ethnicity, income level and geography. IDF, International Diabetes Federation; IGT, impaired glucose tolerance; m, million

The complexities of prediabetes identification, described above, can make it challenging to gain on overview of relative prediabetes prevalence from the literature. However, the IDF have published a comprehensive picture of the current and future trends of prediabetes prevalence based on IGT in individuals aged 20–79 years (Fig. 1) [9]. The global prevalence of IGT was estimated at 7.3% of the adult population in 2017, equivalent to 352.1 million individuals. By 2045 the prevalence is anticipated to increase to 8.3% of the global adult population, equivalent to an estimated 587 million individuals. There is no significant difference of prevalence in men and women, and around half of all individuals with IGT are aged under 50 years [9]. Unadjusted regional prevalence is currently highest in the North America and Caribbean (15.4%) and Central and South America (10.0%) IDF regions, and lowest in the South East Asia (3.0%) and European (5.5%) regions [9]. It should be noted that these estimates are based on IGT only, so prevalence may be higher if additional criteria were taken into consideration.

## Factors that affect prevalence rates of prediabetes

A number of epidemiological studies have demonstrated a clear relationship between ethnicity and likelihood of prediabetes; African American, Native American, South Asian and Hispanic have all been shown to have an increased risk of having prediabetes compared with their Caucasian counterparts [14, 24].

A complex interaction of further factors that include life expectancy, socioeconomic status, wealth, access to healthcare services, levels of education, exposure to disease/public health awareness initiatives, and regional levels of obesity influence prevalence rates [25–27]. As populations become more urbanised, become wealthier, gain better access to nutrition, healthcare and education, and live longer, rates of prediabetes are expected to increase. These increases are expected to be more pronounced in developing rather than in developed countries as lifestyles become more ‘westernised’.

## Conclusions

The reported prevalence for prediabetes vary widely within the literature, reflecting the heterogeneity of both the methods used to define the condition, and of the characteristics of the populations themselves. What is clear, however, is that the prevalence of prediabetes is increasing rapidly in all parts of the world. Action is required to halt this increase, and to avoid the future diabetes epidemic that currently threatens to overwhelm global healthcare provisions.

## Abbreviations

ADA: American Diabetes Association
CKD: Chronic kidney disease
DPP: Diabetes Prevention Program
DPPOS: Diabetes Prevention Program Outcomes Study
FPG: Fasting plasma glucose
HbA_1C_: Glycated haemoglobin
ICD-10-CM: International Classification of Diseases, 10th Revision, Clinical Modification
IDF: International Diabetes Federation
IFG: Impaired fasting glucose, IGT, impaired glucose tolerance
m: Million
ND: Not defined
OGTT: Oral glucose tolerance test
PG: Plasma glucose
T2DM: Type 2 diabetes
WHO: World Health Organization

## References

[1] Tabak AG, Herder C, Rathmann W, Brunner EJ, Kivimaki M (2012). Prediabetes: a high-risk state for diabetes development. Lancet.

[2] Diabetes Prevention Program Research Group (2007). The prevalence of retinopathy in impaired glucose tolerance and recent-onset diabetes in the diabetes prevention program. Diabet Med.

[3] Lee CC, Perkins BA, Kayaniyil S, Harris SB, Retnakaran R, Gerstein HC, Zinman B, Hanley AJ (2015). Peripheral neuropathy and nerve dysfunction in individuals at high risk for type 2 diabetes: the PROMISE cohort. Diabetes Care.

[4] Echouffo-Tcheugui JB, Narayan KM, Weisman D, Golden SH, Jaar BG (2016). Association between prediabetes and risk of chronic kidney disease: a systematic review and meta-analysis. Diabet Med.

[5] Huang Y, Cai X, Mai W, Li M, Hu Y (2016). Association between prediabetes and risk of cardiovascular disease and all cause mortality: systematic review and meta-analysis. Bmj.

[6] Metzger BE, Lowe LP, Dyer AR, Trimble ER, Chaovarindr U, Coustan DR, Hadden DR, McCance DR, Hod M, McIntyre HD (2008). Hyperglycemia and adverse pregnancy outcomes. N Engl J Med.

[7] ICD-10-CM codes [https://www.icd10data.com].

[8] Camila Furtado de Souza, Jorge Luiz Gross, Fernando Gerchman, Piglet CB: pre-diabetes: diagnosis, evaluation and treatment of chronic complications**.** Arq Bras Endocrinol Metab 2012, 56. 10.1590/s0004-27302012000500001 22911279

[9] International diabetes federation: IDF diabetes atlas - 8th edition**,** 2017**.** 35914061

[10] 2017 IDF: IDF clinical practice recommendations for managing type 2 diabetes in primary care**.** 10.1016/j.diabres.2017.09.002 28962686

[11] American Diabetes Association. 3. Prevention or delay of type 2 diabetes: standards of medical Care in Diabetes-2019. Diabetes Care. 2019, 42:S29–33. 10.2337/dc19-S003 30559229

[12] Centers for Disease Control and Prevention; https://www.cdc.gov/diabetes/data/statistics-report/prevalence.html

[13] American Diabetes Association. 2. Classification and diagnosis of diabetes: standards of medical Care in Diabetes-2019. Diabetes Care. 2019, 42:S13–28. 10.2337/dc19-S002 30559228

[14] Yip WCY, Sequeira IR, Plank LD, Poppitt SD. Prevalence of pre-diabetes across ethnicities: a review of impaired fasting glucose (IFG) and impaired glucose tolerance (IGT) for classification of Dysglycaemia. Nutrients. 2017;9. 10.3390/nu9111273 PMC5707745 29165385

[15] Sequeira IR, Poppitt SD: HbA1c as a marker of prediabetes: a reliable screening tool or not? *Insights Nutr Metabol* 2017, 1**:**21–29.

[16] Yudkin JS, Montori VM (2014). The epidemic of pre-diabetes: the medicine and the politics. Bmj.

[17] Barry E, Roberts S, Oke J, Vijayaraghavan S, Normansell R, Greenhalgh T. Efficacy and effectiveness of screen and treat policies in prevention of type 2 diabetes: systematic review and meta-analysis of screening tests and interventions. BMJ. 2017;356. 10.1136/bmj.i6538 28052845

[18] Herman WH, Ma Y, Uwaifo G, Haffner S, Kahn SE, Horton ES, Lachin JM, Montez MG, Brenneman T, Barrett-Connor E (2007). Differences in A1C by race and ethnicity among patients with impaired glucose tolerance in the diabetes prevention program. Diabetes Care.

[19] Knowler WC, Fowler SE, Hamman RF, Christophi CA, Hoffman HJ, Brenneman AT, Brown-Friday JO, Goldberg R, Venditti E, Nathan DM (2009). 10-year follow-up of diabetes incidence and weight loss in the diabetes prevention program outcomes study. Lancet.

[20] Knowler WC, Barrett-Connor E, Fowler SE, Hamman RF, Lachin JM, Walker EA, Nathan DM (2002). Reduction in the incidence of type 2 diabetes with lifestyle intervention or metformin. N Engl J Med.

[21] Diabetes Prevention Program Research Group (2015). HbA1c as a predictor of diabetes and as an outcome in the diabetes prevention program: a randomized clinical trial. Diabetes Care.

[22] Karve A, Hayward RA (2010). Prevalence, diagnosis, and treatment of impaired fasting glucose and impaired glucose tolerance in nondiabetic U.S. adults. Diabetes Care.

[23] Blum J, Aeschbacher S, Schoen T, Bossard M, Pumpol K, Brasier N, Risch M, Risch L, Conen D (2015). Prevalence of prediabetes according to hemoglobin A1c versus fasting plasma glucose criteria in healthy adults. Acta Diabetol.

[24] Sentell TL, He G, Gregg EW, Schillinger D (2012). Racial/ethnic variation in prevalence estimates for United States prediabetes under alternative 2010 American Diabetes Association criteria: 1988-2008. Ethn Dis.

[25] Anjana RM, Deepa M, Pradeepa R, Mahanta J, Narain K, Das HK, Adhikari P, Rao PV, Saboo B, Kumar A (2017). Prevalence of diabetes and prediabetes in 15 states of India: results from the ICMR-INDIAB population-based cross-sectional study. Lancet Diabetes Endocrinol.

[26] Yisahak SF, Beagley J, Hambleton IR, Narayan KM (2014). Diabetes in North America and the Caribbean: an update. Diabetes Res Clin Pract.

[27] Akter S, Rahman MM, Abe SK, Sultana P (2014). Prevalence of diabetes and prediabetes and their risk factors among Bangladeshi adults: a nationwide survey. Bull World Health Organ.

